# DQRNet: Dynamic Quality Refinement Network for 3D Reconstruction from a Single Depth View

**DOI:** 10.3390/s25051503

**Published:** 2025-02-28

**Authors:** Caixia Liu, Minhong Zhu, Haisheng Li, Xiulan Wei, Jiulin Liang, Qianwen Yao

**Affiliations:** 1 Beijing Key Laboratory of Big Data Technology for Food Safety, School of Computer and Artificial Intelligence, Beijing Technology and Business University, No. 33, Fucheng Road, Haidian District, Beijing 100048, China; liucaixia@btbu.edu.cn (C.L.); 2230702042@st.btbu.edu.cn (M.Z.); 2431062109@st.btbu.edu.cn (J.L.); 2431061063@st.btbu.edu.cn (Q.Y.); 2 School of Logistics, Beijing Wuzi University, No. 321, Fuhe Street, Tongzhou District, Beijing 101149, China; weixiulan@bwu.edu.cn

**Keywords:** 3D shape completion, dynamic encoder–decoder, global and local point refiners, single depth view

## Abstract

With the widespread adoption of 3D scanning technology, depth view-driven 3D reconstruction has become crucial for applications such as SLAM, virtual reality, and autonomous vehicles. However, due to the effects of self-occlusion and environmental occlusion, obtaining complete and error-free 3D shapes directly from 3D scans remains challenging, as previous reconstruction methods tend to lose details. To this end, we propose Dynamic Quality Refinement Network (DQRNet) for reconstructing complete and accurate 3D shape from a single depth view. DQRNet introduces a dynamic encoder–decoder and a detail quality refiner to generate high-resolution 3D shapes, where the former designs a dynamic latent extractor to adaptively select important parts of an object and the latter designs global and local point refiners to enhance the reconstruction quality. Experimental results show that DQRNet is able to focus on capturing the details at boundaries and key areas on ShapeNet dataset, thereby achieving better accuracy and robustness than SOTA methods.

## 1. Introduction

Over the past few years, the rise in use of 3D scanning technology has led to major advancements in 3D sensing applications, including SLAM, virtual reality, and autonomous vehicles [1,2,3,4,5,6]. Due to self-occlusion and environmental occlusion, obtaining perfect 3D shapes directly from a 3D scanner is not easy in the real world, and the obtained 3D shapes often miss bits, which can mess up how well it works for applications. Therefore, 3D reconstruction based on depth images is becoming more and more important [7]. For different applications, the main representations of 3D shapes contain point clouds [8], meshes [9,10], and 3D voxels [11,12]. As a regular structure, voxels are a straightforward generation of pixels to 3D cases, and are easily fitted into deep networks which are popular for generative 3D tasks and worthy of further exploration [13].

Conventional 3D reconstruction methods often use 3D retrieval-based techniques [14,15], Poisson surface reconstruction [16], and database fitting [17] to recover missing parts of objects and fill in existing holes. However, these methods generally yield sparse 3D shapes lacking in fine details, need an extensive amount of images, and rely on crafted algorithms for feature extraction [18]. To tackle these problems, researchers have come up with deep learning models which mine valuable patterns from a large amount of data to fill in the missing parts [19,20,21,22,23,24]. These learning-based methods usually use an autoencoder and try to rebuild the whole shape by making the predicted shapes as close as possible to the full shapes. However, due to the interference background of objects and the noise generated during data processing, the reconstruction quality is poor. Meanwhile, the reconstruction results often lose details due to self-occlusion and significant shape variations among different categories of objects.

To address the above challenges, we propose Dynamic Quality Refinement Network (DQRNet), which can reconstruct a fine-grained 3D shape with high-resolution and detailed boundaries from a single depth view. DQRNet first converts a single depth view into a 2.5D voxel grid and feeds it into an encoder–decoder network to reconstruct a coarse 3D shape with the resolution of $256^{3}$, where DQRNet introduces a dynamic latent extractor between the encoder and decoder to avoid the interference from noisy features and enhancing the robustness. The coarse prediction is then fed to a detail quality refiner consisting of a global point refiner and a local point refiner, which guide the generation of reasonable 3D shapes by enhancing both global and local points. The global point refiner adopts a discriminator based on multi-head self-attention, which helps it to focus on different levels of global features, thereby improving details by fully capturing long-distance dependencies of objects. The local point refiner samples points with higher partial instability, extracts multilevel local and nonlocal features of these points, then utilizes a weighted fusion network to adaptively combine the pre- and post-predicted points to obtain boundary details of objects.

Our contributions are as follows:

- DQRNet introduces a dynamic encoder–decoder, which adopts a dynamic latent extractor to select the most valuable latent information. This can reduce noise and enhance robustness.
- DQRNet introduces a detail quality refiner to enhance the predictions, which consists of global point refiner and local point refiner. The global refiner employs a discriminator based on multi-head self-attention to update global information. The local refiner combines the pre- and post-judgments by weighted fusion, which helps to refine the details at the boundaries.
- DQRNet improves the average IoU by 2.09% and average CE by 2.88% on the ShapeNet dataset.

## 2. Related Work

**Single RGB View for 3D Reconstruction.** The challenge of creating three-dimensional representations from two-dimensional images is a fundamental issue with extensive applications in the field of computer vision. The most common way to tackle this involves presenting it as a predictive challenge in which a data-driven model is taught to generate a three-dimensional representation from two-dimensional image input. Many deep learning techniques have attempted this by predicting various types of 3D shapes, including meshes [25,26,27,28], point clouds [29,30,31], and neural implicit fields [32,33,34]. While these methods have had some success, they often have trouble creating detailed 3D models of complex objects because there is a great deal of uncertainty about what is in the parts of the object that are not visible in the 2D image. These regression-based methods are limited in that they cannot really deal with the uncertainty in single-view reconstruction. To mitigate this limitation, recent works [35,36] have explored global context modeling using transformers. While these methods improve reconstruction quality, they still lack fine-grained details due to the limited information available from a single view.

**Single Depth View for 3D Reconstruction.** As depth sensors have improved, depth images are also being used to reconstruct the structure of objects. Depth images provide explicit geometric information, reducing the ambiguity present in RGB-based methods. Several works [17,37,38,39,40] have developed methods that can predict 3D shapes with higher resolution. Zhao et al. [41] proposed 3D-RVP as an uncomplicated but effective approach for accurately reconstructing a complete 3D shape from a single depth view. Yang et al. [24] took a different approach by reconstructing shapes from one depth view using a framework which includes an adversarial component for enhancing the quality of the reconstruction. Aboukhadra et al. [42] introduced a groundbreaking approach to reconstructing the shapes of hands and objects. Liu et al. [13] introduced a spatial relationship preserving adversarial network for detailed 3D reconstruction from a single depth image, transitioning from a rough outline to fine details. Despite these advances, existing depth-based methods can still exhibit inaccuracies in structure and geometry. This is primarily because these techniques rely heavily on convolutional layers that are limited to capturing local information. To achieve a more accurate structure from incomplete shape, it is crucial to consider the nonlocal relationships between different parts of the object. Unfortunately, this aspect has not been adequately addressed in previous research on 3D shape completion from single depth view. Our work overcomes this limitation by introducing a dynamic encoder–decoder that adaptively selects important latent features, thereby reducing noise interference and improving reconstruction fidelity.

**Deep Generative Models for 3D Reconstruction.** Generative models have been widely explored for 3D shape synthesis, contributing to both shape reconstruction and detail refinement. In the last few years, there have been many studies on creating 3D shapes, and current 3D generative models are based on different structures. These include Generative Adversarial Networks (GANs) [43,44,45,46], Variational Autoencoders (VAEs) [19,47,48,49], normalizing flows [19,50], autoregressive models [51], and energy-based models [52]. GAN-based methods employ adversarial training to generate plausible 3D shapes. However, GAN-based models often suffer from mode collapse, leading to limited shape diversity. VAEs and normalizing flow models aim to learn structured latent spaces for shape generation, but often lack high-resolution details. Inspired by the success of 2D latent diffusion models [53], researchers have applied diffusion models to 3D shape generation [36,54,55]. These methods improve shape quality but require high computational resources. Following the the performance of latent diffusion-based models [53] in producing 2D images, a number of studies [56,57,58] have utilized generative modeling in the latent space for 3D shapes. This aspect aims to decrease computational demands and improve the quality of the generated 3D structures. Our DQRNet differs from these generative models by its incorporation of dynamic refinement reconstruction. Rather than learning a latent shape distribution from scratch, DQRNet enhances coarse reconstructions by means of a structured latent extractor and a multistage quality refinement mechanism. Recently, Neural Radiance Fields (NeRF) have become popular for 3D scene rendering and novel view synthesis. However, NeRF-based methods differ fundamentally from our DQRNet in both 3D representation and task objectives. NeRF represents 3D information as an implicit radiance field, encoding scene geometry and appearance within a neural network. In contrast, DQRNet explicitly reconstructs 3D shapes using voxel representations and generates the complete 3D occupancy grid from a partial observation. In this paper, we focus on comparing methods that share the same explicit 3D shape representation.

## 3. DQRNet

### 3.1. Overview

**Figure 1:**
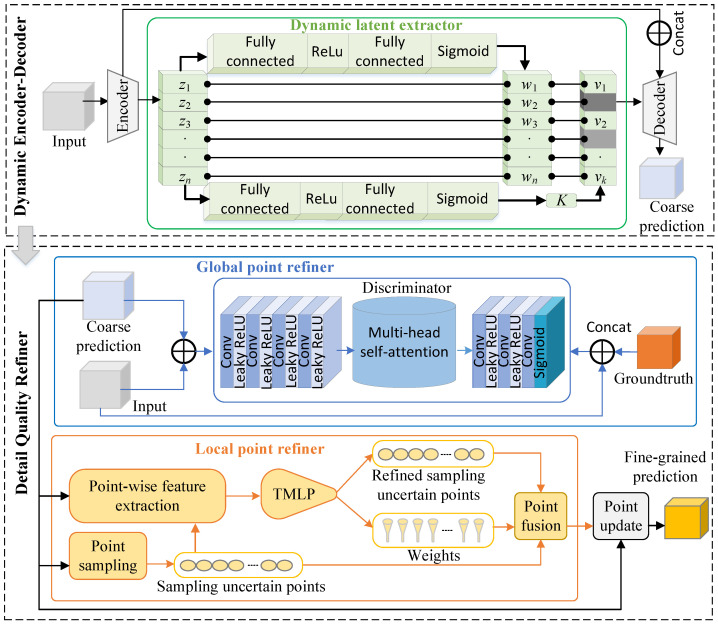
The architecture of our proposed DQRNet. DQRNet introduces a dynamic encoder–decoder to obtain a coarse prediction and multiscale features from a single depth view. DQRNet then utilizes global and local point refiners to enhance the reconstruction quality and achieve a fine-grained 3D shape.

As shown in Figure 1, DQRNet performs dense reconstruction while preserving the boundary details from a single depth view of an object. DQRNet contains two parts: a dynamic encoder–decoder network, and a detail quality refiner consisting of a global point refiner and a local point refiner. DQRNet utilizes the dynamic encoder–decoder network to select the most valuable latent information from a single depth view (see Section 3.2), then introduces a global point refiner to capture long-distance dependencies of objects, thereby improving the overall global structure (see Section 3.3.1). The local point refiner samples points with higher instability, extracts multilevel features, and utilizes a weighted fusion network to adaptively combine pre- and post-predicted points, thereby refining the boundary details (see Section 3.3.2). By integrating these components, DQRNet effectively enhances the 3D shape reconstruction quality, achieving improved IoU and Chamfer error on the ShapeNet dataset. In this paper, the 3D shape of an object is expressed as the probability distribution of binary variables on a 3D voxel grid, where 1 indicates that the voxel is occupied and 0 indicates that the voxel is unoccupied.

A few notations are introduced as follows: scalars, vectors, tensors, and functions are respectively denoted by non-bold italic letters, bold lowercase letters, bold uppercase letters, and calligraphic uppercase letters letters.

### 3.2. Dynamic Encoder–Decoder

#### 3.2.1. Encoder–Decoder

This part is composed of an encoder–decoder with a skip connection and an upsampling component. Standard encoder–decoder architectures often tend to compress important structural details into a latent space, leading to loss of fine-grained features. Inspired by [59], our encoder–decoder is designed as a residual structure. The encoder extracts multiscale features while suppressing noise via residual blocks. The decoder leverages the skip connection to integrate local structural details with global shape priors, enabling plausible completion of occluded regions.

Specifically, the encoder consists of 18 layers. The first layer is a convolution operation with 64 channels, while the subsequent 16 layers are a residual network with four blocks. Each block consists of four convolutional layers with $3^{3}$ kernels. The strides are set to $2^{3}$, $1^{3}$, $1^{3}$, and $1^{3}$, respectively, followed by a LeakyReLU activation function. The channels are doubled at each block. The final layer is a fully connected layer that outputs a 2000-dimensional latent vector. The decoder is composed of four symmetric up-convolutional blocks. Each block consists of four transpose convolution layers with kernel sizes of $3^{3}$ and strides of $1^{3}$, except for the first layer, which uses a stride of $2^{3}$. Each layer is followed by a ReLU activation function. The decoder finally outputs a $64^{3}$ shape. The upsampling component comprises two up-convolutional layers, which increase the resolution of the shape to $256^{3}$.

#### 3.2.2. Dynamic Latent Extractor

In a standard encoder–decoder model, the latent space of an object is represented as a vector in which each element has the same weight. However, in real-world environments not all latent features are equally relevant due to the presence of noise and redundant information. These irrelevant features can result in diminished quality of the reconstructed model. To tackle this issue, a dynamic latent extractor is introduced to select and preserve the relevant latent features. The implementation details are shown in Algorithm 1, which consists of two neural networks W(·) and K(·) having the same structure. However, the numbers of nodes in the two fully connected layers of the former are 2000 and 2000, respectively, while those of the latter are 1000 and 1, respectively. The feature vector $z=\left\{z^{1},z^{2},\cdots ,z^{i},\cdots ,z^{n}\right\}\in R^{n}$ from the above encoder is fed into the neural network W(·) to predict the weight of each latent code $z^{i}$, represented as W(z)=w, where the network contains two fully connected layers. They adopt a ReLU and sigmoid activation function, respectively. Similarly, the latent vector is fed into another neural network K(·) to predict a scalar *k*, denoted as K(z)=k. Here, *k* is a dynamic parameter that is used to select the relevant latent features. A function T(·) is then introduced to obtain the *k*th value $w^{*}$ among the top *k* values of the w, sorted in descending order. In this way, we obtain a new vector $\bar{w}$ by Equation (1). Note that we maintain the positional consistency between the latent vector v and z, ensuring that the corresponding features are mapped to the correct positions:

(1) $${\bar{w}}^{i}=\left\{\begin{array}{cc} 1, & \mathrm{if}\;w^{i}>w^{*}\\ 0, & \mathrm{otherwise} \end{array}\right.\;i=1,2,\cdots ,n$$

(2) $$v=z⊙\bar{w}$$

where $v=\left\{v^{1},0,v^{2},\cdots ,0,\cdots ,v^{k}\right\}\in R^{n}$ represents the latent vector after dynamic selection and ⊙ denotes the element-wise multiplication. By suppressing latent features with low relevance, the negative impact of noise on reconstruction quality can be reduced. In addition, the dynamic latent vector is also used as a strong sparse regularization to avoid over-fitting certain features, which helps to improve the robustness of the model.
**Algorithm 1:** Dynamic latent extractor  **Input**   : Latent vector $z=z^{1},z^{2},\ldots ,z^{i},\ldots ,z^{n}\in R^{n}$ from the encoder  **Output**: Latent vector $v=v^{1},0,v^{2},\ldots ,0,\ldots ,v^{k}\in R^{n}$     //*Step 1: Predict the weights*  1 **Initialization:**
$\bar{w}\leftarrow 0$  2 w←W(z)     //*Step 2: Predict a threshold to retain the top k latent features*  3 k←K(z)     //*Step 3: Generate binary masks by retaining the top k dimensions*  4 $w^{*}\leftarrow T\left(w\right)$//*Obtain the kth value among the top k values in the descending sorted*
w

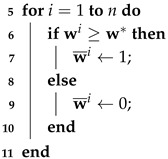

     //*Step 4: Refine the latent vector by element-wise multiplication* 12  $v\leftarrow z⊙\bar{w}$ by Equation (2) //*Preserve the original positional mapping* 13 **return **v

### 3.3. Detail Quality Refiner

To enhance the points with high instability in the above coarse predictions, a detail quality refiner is developed. The detail quality refiner consists of a global point refiner and a local point refiner. The former leverages nonlocal information to improve the overall structure, while the latter selectively refines uncertain regions to obtain the boundary details of shape.

#### 3.3.1. Global Point Refiner

To enhance the authenticity and realism of the predicted 3D shapes, the global point refiner is designed as a discriminator with seven 3D convolutional layers and 3D multi-head self-attention (MSA). Each convolutional layer employs $4^{3}$ filters with strides of $2^{3}$ followed by a LeakyReLU activation function, except for the final layer, which uses a sigmoid activation function. The number of output channels for these convolutional layers starts at 16 and doubles with each subsequent layer, ending up with 1024. The MSA can learn relevant clues from nonlocal regions in order to capture multilevel global information and improved details. The initial convolutional layer merges the coarse 3D shape (i.e., a 3D voxel grid of $256^{3}$) and input 2.5D depth view (i.e., a 2.5D voxel grid of $64^{3}$) as the fake input, then merges the ground truth 3D shape (i.e., a 3D voxel grid of $256^{3}$) and input 2.5D depth view as the real input. The global point refiner finally outputs fake and real discriminative feature vectors with sizes of 8192.

#### 3.3.2. Local Point Refiner

The main source of reconstruction errors often lies in regions with high uncertainty. These uncertain regions primarily arise at object boundaries due to occlusions and missing data. To address this problem, we introduce local point refiner. The implementation details are shown in Algorithm 2. The local point refiner has four stages: a point sampling module, a point-wise feature extraction module, two multilayer perceptron (TMLP) networks, and a point fusion module.
**Algorithm 2:** Local point refiner
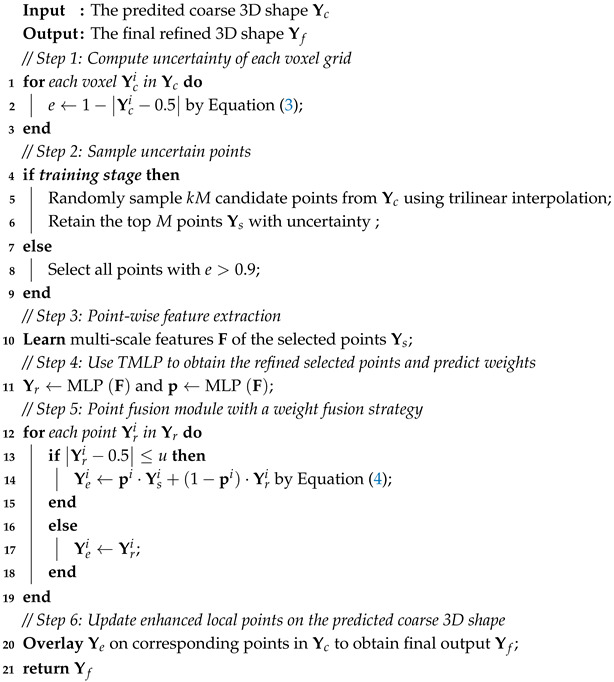


The point sampling process seeks to find points with high uncertainty in the coarse prediction. Each predicted voxel has an occupied probability. The closer the probability is to 0.5, the higher its uncertainty. The uncertainty *e* is obtained by the following Equation (3):

(3) $$e=1-\left|Y_{c}^{i}-0.5\right|$$

where $Y_{c}^{i}$ is the occupied probability of the *i*th voxel for the predicted coarse shape $Y_{c}$.

With the aim of accelerating training and enhancing the generalization ability, we adopt different point sampling strategies in the training and inference stages. In the training stage, we sample kM candidate points from the coarse prediction using trilinear interpolation based on randomly generated coordinates of a uniform distribution, where *k* determines the inclination towards regions with high uncertainty. We then select *M* points with the highest uncertainty from the candidate points, where *M* and *k* are empirically set to 65536 and 4 in this experiment, respectively. During inference, we sample the points with uncertainty e>0.9 in the experiments.

The point-wise feature extraction process is responsible for learning more rich features of the selected points. For any selected point, its occupancy probability can be extracted from this coarse output. We obtain the multiscale features of the selected points by performing trilinear interpolation on both fine-grained features and coarse-grained features. The former are taken from the first layer and the first block of the encoder, while the latter are taken from the last two blocks of the decoder. The fine-grained features contain more local information, enabling the model to capture complex details, while the coarse-grained features contain more global information, helping to provide broader context cues.

The TMLP networks aim to refine the sampled uncertain points and predict their weights based on the point-wise features obtained in the previous step. This allows the model to adaptively control the influence of refined points, preventing overcorrection. A TMLP network consists of two structurally consistent MLP networks. One MLP outputs the refined predictions for each selected point, while the other MLP outputs their adaptive weights. Each MLP network consists of four layers; the first layer has 152 nodes, corresponding to the dimensionality of the point-wise features, while the two hidden layers have 100 and 50 nodes, respectively, and the last layer has one node. Each layer is followed by a ReLU activation function, except for the last layer, which uses a sigmoid activation function.

The point fusion module is designed to fuse the sampled uncertain points $Y_{s}$ and refined uncertain points $Y_{r}$ using the predicted weights p from the TMLP. To reduce unnecessary computational overhead, we apply a selective weighted fusion strategy to low-confidence points only, while keeping the high-confidence points unchanged. Specifically, we compute the confidence of the points $Y_{r}$ using Equation (3). To refine points with low confidence while retaining points with high confidence, we set a threshold *u*. As shown in Equation (4), we retain those points with higher confidence than *u* from the output of the TMLP, and design a weight fusion strategy to deal with the points with a lower confidence than *u*. The comparison results of different values of *u* are shown in ablation studies. The tables are 6 and 7. If it is not a, I have revised it as ablation studies. As a result, the local point refiner outputs the enhanced points $Y_{e}$.

(4) $$Y_{e}^{i}=\left\{\begin{array}{cc} p^{i}Y_{s}^{i}+\left(1-p^{i}\right)Y_{r}^{i} & \;\mathrm{if}\;\left|Y_{r}^{i}-0.5\right|\leq u\\ Y_{r}^{i} & \;\mathrm{if}\;\left|Y_{r}^{i}-0.5\right|>u \end{array}\right.$$

Finally, we overlay the enhanced local points $Y_{e}$ on the coarse shape $Y_{c}$ to obtain the final output $Y_{f}$.

## 4. Loss Function

Our loss function consists of four parts: the dynamic encoder–decoder loss ($L_{c}$), local point refiner loss ($L_{l}$), global point refiner loss ($L_{g}$ and $L_{d}$), and fine-grained prediction loss ($L_{f}$). For the dynamic encoder–decoder loss ($L_{c}$) and fine-grained prediction loss ($L_{f}$), we utilize a modified binary cross-entropy loss. This loss function includes a penalty parameter that increases the penalty when a voxel with a value of 1 is mistakenly identified as 0. The losses are expressed as follows:

(5) $$L_{c}=-\frac{1}{N}\sum_{i=1}^{N}\left[\lambda Y^{i}\log Y_{c}^{i}+\left(1-\lambda \right)\left(1-Y^{i}\right)\log\left(1-Y_{c}^{i}\right)\right]$$

(6) $$L_{f}=-\frac{1}{N}\sum_{i=1}^{N}\left[\lambda Y^{i}\log Y_{f}^{i}+\left(1-\lambda \right)\left(1-Y^{i}\right)\log\left(1-Y_{f}^{i}\right)\right]$$

where *N* represents the number of voxels in a voxel grid, $Y_{c}^{i}\in \left(0,1\right)$ and $Y_{f}^{i}\in \left(0,1\right)$ are the occupied probability of the *i*th voxel for predicted coarse and fine-grained shapes, respectively, $Y^{i}\in \left[0,1\right]$ is the corresponding ground truth, and λ is used to balance the weights for predicting occupied and unoccupied voxels in the experiments.

The global point refiner loss consists of two parts, the generator loss $L_{g}$ and the discriminator loss $L_{d}$:

(7) $$L_{g}=-E\left[D\left(Y_{c}|X\right)\right]$$

(8) $$\begin{array}{cc} L_{d} & =E\left[D\left(Y_{c}|X\right)\right]-E\left[D\left(Y|X\right)\right]+\eta E\left[{\left(∥\nabla_{{\hat{Y}}_{c}}D\left({\hat{Y}}_{c}|X\right){∥}_{2}-1\right)}^{2}\right] \end{array}$$

where ${\hat{Y}}_{c}=\epsilon Y+\left(1-\epsilon \right)Y_{c},\epsilon ∼U\left[0,1\right]$, X is the input depth view, $Y_{c}$ is the predicted coarse shape, Y is the corresponding ground truth, and *D* is the discriminator. The weight η is a commonly used regularization parameter that controls the balance between optimizing the gradient penalty and the original objective in WGAN-GP [60] to ensure stable training.

The local point refiner loss ($L_{l}$) is used to punish the sampling of uncertain points in the predicted coarse shape, and a cross-entropy loss function is employed. This loss can help the model to concentrate on learning the detailed and local information in a voxel grid, and is expressed as follows:

(9) $$L_{l}=-\frac{1}{M}\sum_{i=1}^{M}\left[\lambda z^{i}\log z_{c}^{i}+\left(1-\lambda \right)\left(1-z^{i}\right)\log\left(1-z_{c}^{i}\right)\right]$$

where *M* is the total number of uncertain sampling points, $z_{c}^{i}$ is the predicted latent representation of the *i*th point for the coarse shape, and $z^{i}$ is the corresponding ground truth obtained by interpolation from the nearest value in a voxel grid.

In summary, the total generative loss $L_{tg}$ and the discriminative loss $L_{d}$ are alternately optimized in the experiments. The loss $L_{tg}$ is expressed as follows:

(10) $$L_{tg}=\lambda \left(L_{c}+L_{f}+\gamma L_{l}\right)+\left(1-\lambda \right)L_{g}$$

where the weights γ and λ are determined through empirical tuning and experimental validation. Considering that the local point refiner loss $L_{l}$ focuses on refining specific regions, it is assigned a relatively low weight of γ=0.1. In contrast, both the dynamic encoder–decoder loss $L_{c}$ and the fine-grained prediction loss $L_{f}$ are aimed at optimizing the overall reconstruction quality, and as such are assigned higher weights to ensure their stronger influence in the training process. Moreover, compared with the losses $L_{c},L_{f},L_{l}$, which contribute to the core 3D completion task, the adversarial loss is assigned a relatively low weight of (1−λ)=0.15. This helps to mitigate the instability introduced by adversarial training while ensuring that the primary completion objectives remain dominant.

## 5. Experimental Results

### 5.1. Dataset

To train DQRNet, a single depth view and its corresponding comprehensive 3D model are essential. We utilize the dataset [24] from the ShapeNet database. The training dataset contains 213 CAD models and comprises four categories: bench, chair, couch, and table. Each model captures 125 different views, with each view corresponding to a 2.5D depth image and its corresponding 3D voxel grid. Therefore, there are 26,625 training pairs for each category. The testing dataset contains 37 CAD models, and like the training dataset has four categories. For each category, two sets are generated. The first set is scanned from the same 125 views, with each category containing 4625 testing pairs. The second set is scanned from 216 different views, with each category containing 7992 testing pairs.

### 5.2. Experimental Setup

All experiments were implemented on an Intel Xeon Platinum 8362 CPU (Intel Corporation, Santa Clara, CA, USA) and NVIDIA GeForce RTX 3090 GPU (NVIDIA Corporation, Santa Clara, CA, USA) with 2.8 GHZ and 64 GB RAM. DQRNet was trained with a batch size of 3. The voxel grid size was set as $N=256^{3}$. The learning rates of the generative loss $L_{tg}$ and discriminator loss $L_{d}$ were set to 0.0006 and 0.002. To train all the parameters of DQRNet, the Adam optimizer [61] was adopted with $\beta_{1}$ = 0.9 and $\beta_{2}$ = 0.999. The weights η, γ, and λ were set as 10, 0.1, and 0.85, respectively.

### 5.3. Metrics

To quantitatively assess the performance of our method, we employed the Intersection over Union (IoU) and Cross-Entropy (CE) loss, both of which are commonly used in 3D shape completion tasks. The IoU quantitatively measures the overlap between the predicted and ground truth shapes. A higher IoU value suggests better shape completion performance. The IoU is defined as follows:

(11) $$\mathrm{IoU}=\frac{\sum_{i=1}^{N}\left[I\left(Y_{p}^{i}>\theta \right)\cdot Y^{i}\right]}{\sum_{i=1}^{N}\left[I\left(Y_{p}^{i}>\theta \right)+Y^{i}\right]}$$

where $Y_{p}^{i}\in \left(0,1\right)$ and $Y^{i}\in \left[0,1\right]$ represent the occupancy values of the *i*th voxel for the predicted and ground truth shapes, respectively, *N* is the total number of voxels in a voxel grid, and θ is a threshold used to binarize the predicted occupancy values. If $Y_{p}^{i}>\theta$, the voxel is considered occupied; otherwise, it is considered empty. Here, we assigned θ a value of 0.5 in our experiments. Finally, $I\left(X\right)$ is an indicator function that maps a condition to a binary value, with 1 if *X* is true and 0 otherwise.

In addition, the CE loss is to further assess the performance of our model by quantifying the difference between the predicted probability distribution and the real distribution. A lower CE loss indicates a better match between the predicted and true voxel occupancy. The CE loss is defined as follows:

(12) $$\mathrm{CE}=-\frac{1}{N}\sum_{i=1}^{N}\left[Y^{i}\log\left(Y_{p}^{i}\right)+\left(1-Y^{i}\right)\log\left(1-Y_{p}^{i}\right)\right].$$

### 5.4. Comparisons with State-of-the-Art Methods

We conducted a comparative analysis with several recent studies that focus on 3D shape reconstruction from single depth images. To ensure fairness and consistency, we utilized the same evaluation datasets and metrics across all studies.

(1) 3D-EPN [17] presents a method based on data that integrates volumetric deep neural networks with 3D shape generation, introduced to reconstruct incomplete 3D shapes.

(2) Varley et al. [62] presented a robotic grasp planning framework utilizing a 3D-CNN trained on a large dataset to enable fast shape completion from 2.5D images. This framework can obtain unseen objects with improved performance.

(3) SeedFormer [63] utilizes patch seeds to capture both global and local information, and incorporates an upsample transformer to incorporate spatial and semantic relationships. For this comparison, we converted the point cloud output from SeedFormer into 3D voxels with a resolution of $256^{3}$.

(4) SnowflakeNet [64] presents snowflake point deconvolution layers, which progressively refine the point cloud. Each layer generates child points by splitting parent points. For this comparison, we voxelized its outputs.

(5) 3D-RecGAN++ [24] is a straightforward and efficient framework that integrates a skip-connected 3D encoder–decoder with adversarial training to produce a compete and detailed 3D structure from a single 2.5D view. The skip connection preserves high-frequency details, while the adversarial training process enhances shape plausibility and fine details.

(6) 3D-RVP [41] is a two-stage approach for reconstructing a complete 3D geometry from a single depth view. It first utilizes an encoder–decoder network to generate a coarse 3D shape as a voxel grid. Then, a point prediction network selectively samples and predicts occupancies of uncertain voxels, iteratively improving the resolution and accuracy of the 3D reconstruction.

**Figure 2:**
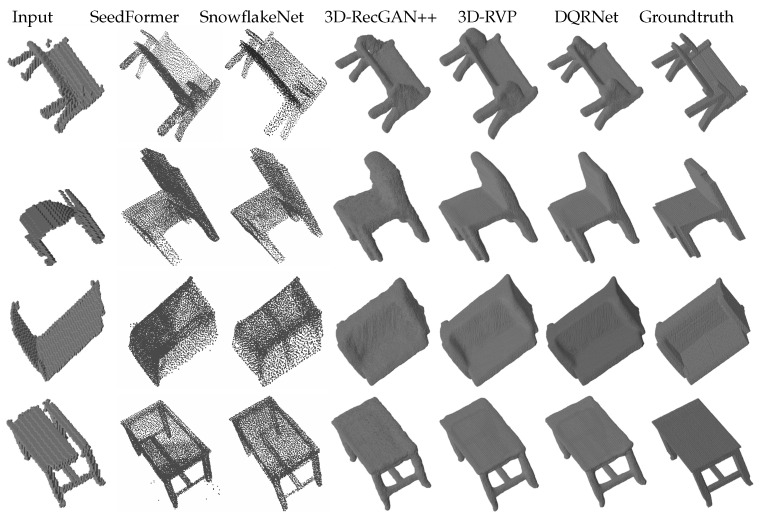
Qualitative results of per-category reconstruction on the testing set with same views.

**Figure 3:**
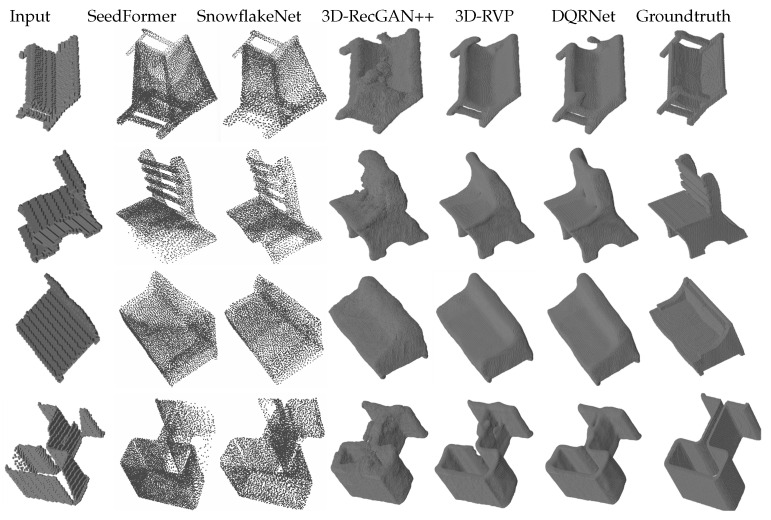
Qualitative results of per-category reconstruction on testing set with the cross views.

We conducted two groups of experiments on the dataset [24]. The first group involved per-category experiments, where the training set and testing set were set as each category. The testing sets consisted of the same 125 views as in the training along with 216 cross views. The results are shown in Table 1 and Table 2 along with Figure 2 and Figure 3. From Table 1 and Figure 2, the results show that DQRNet achieves the best relative performance on the reconstruction of the same views in terms of both IoU and CE loss; compared to 3D-RVP, the average IoU improves by 1.26%, while the average CE loss is reduced by 4.66%. From Table 2 and Figure 3, the results show that DQRNet also attains the best IoU on the reconstruction of the cross views for all categories, along with the best CE for three categories. The average IoU of DQRNet improves by 2.23% compared to 3D-RVP, while the CE loss is reduced by 6.83%. From the qualitative results in Figure 2 and Figure 3, Seedformer tends to cause fragmented shapes, whereas DQRNet produces more complete results and retains finer details. SnowflakeNet only outputs a point cloud with a resolution of 8192 points, and its point-based representation lacks delicate structures; for example, the ends of chair legs often appear incomplete or overly smoothed. In contrast, DQRNet enables more precise reconstruction, effectively capturing both global structure and fine-grained details, leading to a more faithful 3D completion. These results demonstrate that DQRNet is able to capture the intricacies of objects, resulting in reconstructions that are not only more comprehensive but also preserve critical features.

**Figure 4:**
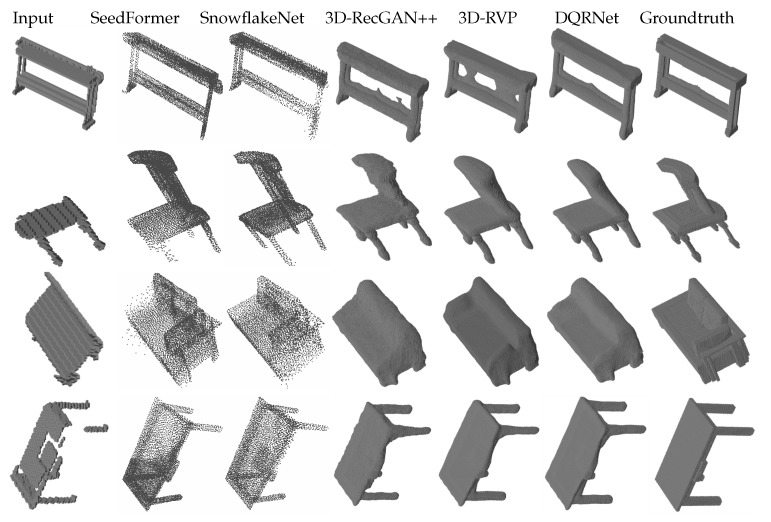
Qualitative results of multi-category reconstruction on testing set with the same views.

**Figure 5:**
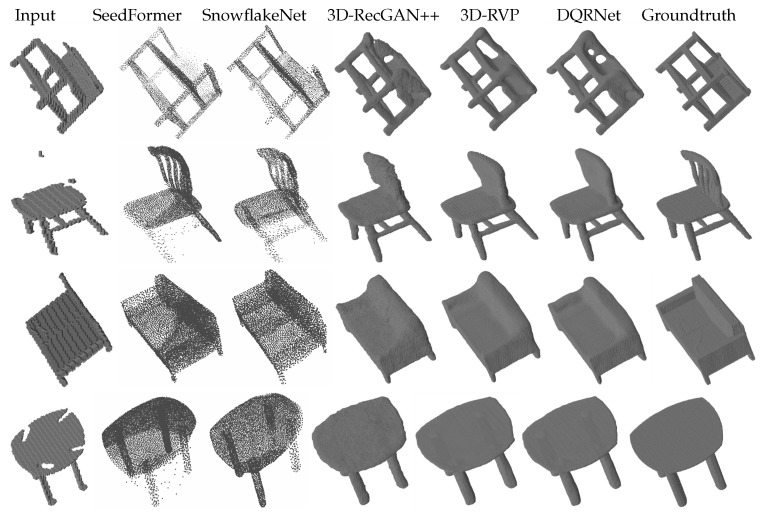
Qualitative results of multi-category reconstruction on the testing set with cross views.

The second group of experiments consisted of multi-category tests. These experiments measure the domain adaptation capability among different categories of 3D objects, with the training set and testing set representing multiple categories. Similar to the first group of experiments, the testing sets consisted of both same and cross views. The results are shown in Table 3 and Table 4 along with Figure 4 and Figure 5. As shown in Table 3, DQRNet achieves the best performance on the same views in terms of both IoU and CE. Notably, DQRNet surpasses 3D-RVP in IoU by an average of 1.77% on the same views, further underscoring the effectiveness of our model. For the bench category, it can be seen from Figure 4 that the other methods often suffer from incomplete shapes, such as missing legs or overly complicated reconstructions. In contrast, DQRNet can accurately reconstruct realistic and plausible shapes, demonstrating its superiority in capturing intricate details. Table 4 demonstrates that DQRNet achieves an impressive average IoU improvement of 3.05% on cross views when compared to 3D-RVP. Figure 5 further demonstrates the robust generalization capability of DQRNet and its ability to preserve local details with remarkable fidelity. These results highlight the superiority of DQRNet in reconstructing multi-category objects from varying views.

From the perspective of views, the same-view results of DQRNet are better the cross-view results. We believe that the same-view settings can make shape reconstruction more reliable and enable more confident voxel occupancy predictions, as the views are known and seen during training. However, the model can only capture limited information when encountering unseen views, making it difficult to predict missing regions of objects. From the perspective of categories, the multi-category results are better than the per-category results. We believe that multi-category training can help the model to generalize better and produce more stable occupancy distributions of objects. In other words, multi-category training generally leads to better performance, as the model is exposed to a more diverse dataset, which helps it to infer missing regions more smoothly. These findings highlight the tradeoffs between specialization and generalization in 3D shape completion, and emphasize the impact of view distribution on model performance.

In addition, we evaluated the computational efficiency of DQRNet in terms of model parameters, floating-point operations (flops), and inference time. DQRNet has 5.5% more parameters (191 M vs. 181 M) and 26.2% more flops (135.41 G vs. 107.29 G) compared with 3D-RVP; however, the inference speed of DQRNet is comparable to that of 3D-RVP. The reason for this is attributed to our local point refiner, which selectively fuses only a subset of points instead of processing all points, thereby reducing unnecessary computations. Moreover, DQRNet delivers superior shape completion results, demonstrating the effectiveness of our design in balancing performance and efficiency.

### 5.5. Ablation Study

We conducted per-category experiments with the same views to validate the effectiveness of the proposed components by progressively adding them to the baseline encoder–decoder network. As presented in Table 5, the quantitative results indicate a significant improvement in both IoU and CE upon introducing the Dynamic Latent Extractor (DLE). This notable enhancement validates the ability of DLE to effectively preserve the most pertinent latent features while mitigating the adverse effects of noise.

Next, we added the Local Point Refiner (LPR) and the Global Point Refiner (GPR) individually. From Table 5, it can be seen that both LPR and GPR contribute to a degree of performance enhancement. Based on these results, GPR can learn more rich global information using the discriminator, while LPR can precisely locate the points that are most likely to be erroneous and re-evaluate them through weighted fusion, which is conductive to more authoritative predictions. By integrating all components, DQRNet achieves the best overall performance, further corroborating the effectiveness of our proposed components.

In addition, we conducted two types of experiments on the uncertainty *e* in Equation (3) and threshold *u* in Equation (4). The comparison results in terms of IoU performance on the bench category with different values of *e* and *u* are shown in Table 6. It can be observed that the reconstruction performance of e>0.9 is better than that of e>0.8. Based on this analysis, when e>0.9, DQRNet is more effective at selecting points with high uncertainty while minimizing changes to points that have already been correctly predicted. This can ensure that the model focuses on refining those points that are most likely to be incorrect. The multi-category comparison results in terms of IoU performance with different *u* are presented in Table 7, considering e>0.9. It can be observed that the performance is better when u=0.30. Our goal is to enhance the reliability of the predicted inaccurate points. As shown in Equation (4), we use the expression $\left|y_{r}-0.5\right|$ to represent the confidence level of the points $y_{r}\in \left(0,1\right)$. When the threshold *u* is set close to 0.5, it includes more high-confidence points, thereby incorporating more accurate points; conversely, setting *u* closer to 0 results in the inclusion of too few points. Therefore, selecting a balanced value of u=0.3 yields better performance.

## 6. Conclusions and Discussion

In this paper, we have proposed a novel framework called DQRNet which can reconstruct missing shapes in 3D scans caused by occlusion problems. By innovatively combining a dynamic latent extractor and detail quality refiner, DQRNet is able to generate complete, high-resolution, and detailed 3D shapes from a single depth view. Our experimental results demonstrate that DQRNet can enhance reconstruction accuracy and robustness, particularly for the boundaries and key regions of 3D shapes.

In the future, we will continue to optimize the performance of DQRNet while exploring practical application scenarios and applying it to more challenging 3D reconstruction tasks. Additionally, we will investigate how to utilize advanced technique such as diffusion models and NeRF in order to achieve more efficient and accurate 3D reconstruction.

## Figures and Tables

**Table 1:** Per-category IoU and CE loss with same views.

Methods	IoU ↑				CE ↓			
	Bench	Chair	Couch	Table	Bench	Chair	Couch	Table
3D-EPN [17]	0.423	0.488	0.631	0.508	0.087	0.105	0.144	0.101
Varley [62]	0.227	0.317	0.544	0.233	0.111	0.157	0.195	0.191
SeedFormer [63]	0.553	0.618	0.740	0.656	0.038	0.065	0.069	0.044
SnowflakeNet [64]	0.562	0.631	0.745	0.659	0.037	0.063	0.068	0.043
3D-RecGAN++ [24]	0.580	0.647	0.753	0.679	0.034	0.060	0.066	0.040
3D-RVP [41]	0.598	0.668	0.760	0.696	0.032	0.060	0.067	0.039
DQRNet	**0.613**	**0.672**	**0.765**	**0.705**	**0.030**	**0.055**	**0.066**	**0.038**

**Table 2:** Per-category IoU and CE loss with cross views.

Methods	IoU ↑				CE ↓			
	Bench	Chair	Couch	Table	Bench	Chair	Couch	Table
3D-EPN [17]	0.408	0.446	0.572	0.482	0.086	0.112	0.163	0.103
Varley [62]	0.185	0.278	0.475	0.187	0.108	0.171	0.210	0.186
SeedFormer [63]	0.508	0.578	0.628	0.603	0.046	0.080	0.120	0.056
SnowflakeNet [64]	0.518	0.589	0.637	0.623	0.045	0.079	0.118	0.055
3D-RecGAN++ [24]	0.531	0.594	0.646	0.618	0.041	0.074	**0.111**	0.053
3D-RVP [41]	0.554	0.621	0.643	0.656	0.037	0.074	0.138	0.047
DQRNet	**0.575**	**0.630**	**0.655**	**0.668**	**0.036**	**0.068**	0.124	**0.044**

**Table 3:** Multi-category IoU and CE loss with same views.

Methods	IoU ↑				CE ↓			
	Bench	Chair	Couch	Table	Bench	Chair	Couch	Table
3D-EPN [17]	0.428	0.484	0.634	0.506	0.087	0.107	0.138	0.102
Varley [62]	0.234	0.317	0.543	0.236	0.103	0.132	0.197	0.170
SeedFormer [63]	0.542	0.613	0.727	0.628	0.036	0.054	0.066	0.045
SnowflakeNet [64]	0.548	0.624	0.736	0.633	0.035	0.053	0.064	0.043
3D-RecGAN++ [24]	0.581	0.640	0.745	0.667	0.030	0.051	0.063	0.039
3D-RVP [41]	0.596	0.655	0.750	0.687	0.029	0.050	**0.062**	**0.035**
DQRNet	**0.613**	**0.667**	**0.755**	**0.699**	**0.028**	**0.050**	0.063	0.037

**Table 4:** Multi-category IoU and CE loss with cross views.

Methods	IoU ↑				CE ↓			
	Bench	Chair	Couch	Table	Bench	Chair	Couch	Table
3D-EPN [17]	0.415	0.452	0.531	4.477	0.091	0.115	0.147	0.111
Varley [62]	0.201	0.283	0.480	0.199	0.105	0.143	0.207	0.174
SeedFormer [63]	0.532	0.583	0.629	0.609	0.040	0.069	0.097	0.052
SnowflakeNet [64]	0.534	0.586	0.631	0.612	0.039	0.068	0.095	0.050
3D-RecGAN++ [24]	0.540	0.594	0.643	0.621	0.038	0.061	**0.091**	0.048
3D-RVP [41]	0.545	0.617	0.661	0.643	0.035	0.058	0.093	0.043
DQRNet	**0.574**	**0.632**	**0.671**	**0.662**	**0.034**	**0.058**	0.092	**0.043**

**Table 5:** Quantitative results of the ablation study. DLE, LPR and GPR denote the dynamic latent extractor, local point refiner, and global point refiner, respectively.

DLE	LPR	GPR	IoU ↑	CE ↓
×	×	×	0.580	0.034
✓	×	×	0.593	0.032
✓	✓	×	0.605	0.030
✓	×	✓	0.596	0.031
✓	✓	✓	**0.613**	**0.030**

**Table 6:** Comparison of IoU performance with different *e* and *u*.

*u*	*e* > 0.8		*e* > 0.9	
	IoU ↑	CE ↓	IoU ↑	CE ↓
0.20	0.613	0.032	0.613	0.030
0.25	**0.614**	**0.032**	0.613	**0.030**
0.30	0.613	0.033	**0.614**	0.031
0.35	0.608	0.034	0.613	0.031
0.40	0.599	0.035	0.612	0.031

**Table 7:** Comparison of IoU performance with different *u*.

*u*	Bench	Chair	Couch	Table
0.20	0.612	0.664	0.753	0.694
0.25	0.613	0.666	0.754	0.697
0.30	**0.613**	**0.667**	**0.755**	**0.699**
0.35	0.612	0.666	0.755	0.699
0.40	0.610	0.663	0.755	0.698

## Data Availability

Details of the data are shown in Section 5.1.
